# Effects of milk fortification with an advanced chelated trace minerals supplement on growth, health, and nutrient digestibility in dairy calves

**DOI:** 10.5713/ab.24.0701

**Published:** 2025-02-27

**Authors:** Hossein Rajaei-Sharifabadi, Hadi Rahmani, Zahra Shokri, Saideh Fakharzadeh, Somayeh Kalanaky, Mohammad Hassan Nazaran, Ahmad Reza Seradj

**Affiliations:** 1 Department of Animal Science, Faculty of Agriculture, Malayer University, Malayer, Iran; 2 Department of Research and Development, Sodour Ahrar Shargh Co., Tehran, Iran; 3 Department of Animal Science, Universitat de Lleida, Lleida, Spain; 4 Agrotecnio CERCA Center, Lleida, Spain

**Keywords:** Advanced Chelate Compounds Technology, Body Measurements, Fecal Consistency, Medical Treatment, Organic Trace Minerals

## Abstract

**Objective:**

Recent advancements in chelate compound technology offer improved bioavailability of trace minerals, potentially enhancing growth performance and reducing disease incidence. Milk fortification is an efficient method to supplement young calves before transition to solid feed. This study evaluated the response of dairy calves to milk fortification with an advanced chelated trace minerals supplement containing Zn, Mn, Cu, Fe, Co, Cr, and Se, each selected for their specific roles in calf development, synthesized using chelate compoundtechnology.

**Methods:**

Forty-two Holstein dairy calves (21 males and 21 females) were randomly assigned to three experimental groups: control (CON, unfortified milk), chelated minerals (BON, milk fortified with chelated trace minerals), and inorganic minerals (IOR, milk fortified with inorganic trace minerals). Calves received their respective milk treatments daily from d 3 until weaning at 70 d of age. Growth performance and body measurements were assessed throughout the experiment, while rectal temperature, fecal consistency, and frequency of medical treatment were recorded until d 21. Blood metabolites and nutrient digestibility were also determined.

**Results:**

BON supplementation resulted in 9% body weight increase (p = 0.027) and improved some body measurements (p<0.05). The calves in BON group showed a lower fecal consistency index (p<0.05) and required fewer medical treatments compared to CON and IOR (p = 0.103). Blood analysis revealed 27% reduction in globulin levels (p = 0.022), 10% increase in albumin levels (p = 0.023) and a 42% elevation in albumin/globulin ratio (p = 0.042) in BON compared to the CON groups. The activities of liver enzymes were higher in IOR than in the BON and CON group.

**Conclusion:**

Milk fortification with advanced chelated trace minerals significantly enhanced growth performance and health indicators in dairy calves, while inorganic sources showed no beneficial effects. These findings establish the superiority of chelated mineral supplementation for early-life dairy calf development;nevertheless, appropriate supplementation procedures require additional experiments.

## INTRODUCTION

The dairy industry faces significant economic challenges due to calf mortality, with global rates ranging from 3% to 10% in major dairy-producing regions [1–3]. These losses primarily stem from digestive and respiratory diseases, particularly during the critical first week of life [4,5]. Managing calf health requires a comprehensive approach, with special emphasis on immune system development through proper mineral nutrition.

Calf growth and survival are influenced by multiple factors, including proper nutrition, disease status, and stress levels [6,7]. Comprehensive management strategies encompassing precise feeding protocols, optimal colostrum delivery, and appropriate trace mineral supplementation are essential for disease prevention and maximizing growth potential [8,9]. These targeted interventions during early life development are particularly crucial as they establish the foundation for lifetime health and productivity, ultimately reducing economic losses associated with calf mortality in dairy operations. Early nutrition plays a pivotal role in immune system development, with trace minerals particularly critical for key immunological processes including neutrophil function, antibody production, and cytokine signaling [7,10,11].

The impact of mineral deficiencies or restricted placental transfer of minerals on neonatal ruminants’ health, growth, and metabolism has been extensively investigated for over four decades [12]. Trace mineral deficiencies have been associated with numerous diseases in calves, such as myopathy or white muscle disease due to selenium deficiency, goiter caused by iodine deficiency, anemia resulting from iron deficiency, and impaired colostrum immunoglobulin transfer and weakened immune system function due to deficiencies in copper, zinc, and selenium. Studies indicated a high prevalence of calf diarrhea in dairy herds lacking adequate trace mineral supplementation [13]. Supplementation with a combination of trace minerals, including selenium, copper, zinc, and manganese, has been shown to enhance neutrophil count, boost glutathione peroxidase activity, and reduce the incidence of diarrhea and pneumonia in calves [11], potentially due to the role of these minerals in regulating gene expression and modulating signaling pathways [14,15]. Furthermore, supplementing newborn calves’ milk with trace minerals such as zinc [16,17] and selenium [18,19] has demonstrated beneficial effects in reducing diarrhea and improving growth performance. Additionally, trace minerals play a crucial role in rumen growth and development. Jacometo et al [20] reported that supplementing the diets of dairy cows in the last month of pregnancy with organic forms of trace minerals, including copper, zinc, manganese, and cobalt, resulted in enhanced skeletal growth and improved innate immune response in their offspring compared to the control group.

Advanced Chelate compounds technology, a patented process for chelating minerals [21], is fundamental for producing various compounds used in fields such as medicine [22], agriculture [23], and animal production. This patented process enhances mineral absorption, reduces antagonism, and improves tissue retention by protecting minerals within an organic acid matrix for superior bioavailability. In the field of animal feed, this technology has been employed to develop a new generation of organic mineral supplements, where several trace minerals are embedded within a specific organic acid complex. Preliminary studies show that this type of organic mineral supplement has higher bioavailability than the inorganic form for lactating cows and can increase milk fat percentage and reduce mastitis symptoms in dairy cows [24]. In poultry, dietary supplements of trace minerals based on this technology have improved growth performance in broiler chickens compared to inorganic sources [25]. One commercial application of this technology is Bonzaplex7, a comprehensive trace minerals supplement containing copper, zinc, selenium, manganese, chromium, iron, and cobalt. The ratio of minerals in this supplement is selected based on their synergistic effects, as well as the specific affinity of each mineral for organic acids within the organic acid matrix. In a study by Mousavi-Haghshenas et al [26], supplementing starter feed with this product improved the growth rate in dairy calves with low birth weight. Additionally, field observations indicate that mixing this product with milk for pre-weaned calves reduced diarrhea symptoms and promoted growth. Although the individual physiological roles of the included minerals and the advantages of this organic acid-based mineral supplement have been well documented in various species, and despite promising results when added to starter feed [25], controlled studies specifically evaluating the effectiveness of milk fortification with this advanced mineral supplement in pre-weaned dairy calves are lacking. Therefore, this study aimed to assess the effectiveness of the advanced chelated trace minerals supplement on growth performance, starter intake, nutrient digestibility, and health parameters of pre-weaned dairy calves. We hypothesized that milk fortification with these bioavailable minerals would enhance immune function and growth performance through improved mineral absorption and utilization, ultimately contributing to more sustainable and profitable dairy calf rearing practices.

## MATERIALS AND METHODS

### Animal care

All procedures involving animals, including housing, feeding, health monitoring, emergency care, and medical management were conducted in accordance with the National Research Council Guide for the Care and Use of Laboratory Animals. The study protocol was reviewed and approved by the Animal Care and Use committee, Department of Animal Science, Malayer University, Malayer, Iran (400/2-3-618, May. 16/ 2021).

### Animals, management, and experimental treatments

This study was conducted at the dairy farm facilities of Pegah-e-Hamadan, Hamadan, Iran. Forty-two newborn Holstein calves (21 males and 21 females) born between September and October 2021 were enrolled in this study. Based on previous research by Davis Rincker et al [27], the sample size calculation indicated that a minimum of 14 calves per preweaning treatment group would be required to detect significant differences in body weight gain with 80% statistical power and a significance level of 0.05. This estimation was also validated using G*Power software (Version 3.1.9.7; Universitat Kiel, Kiel, Germany). The experiment lasted until December 2021, when all calves were weaned at 70 d of age. Within 2 h after birth, calves were separated from their dams, housed in individual indoor pens (1×2 m), and received colostrum (at least 4 L within the first 24 h) via nipple bottle. From d 3 of age until weaning, the calves were transferred to outdoor individual pens (1×3 m), allowing visual and auditory interaction while preventing physical contact. The pens were cleaned and re-bedded regularly to ensure hygiene, and consistent care was provided throughout the study to ensure a healthy environment that supported the calves’ well-being and development. The calves were fed milk from buckets twice daily at 7:30 AM and 4:00 PM, with volumes of 3, 6, and 4 L/d provided from d 2 to d 14, d 15 to d 50, and d 51 to d 63, respectively. To facilitate the weaning process, 2 L of milk were offered daily from d 64 until completely weaned at d 70 of age. Water and starter feed (Table 1) were offered ad libitum throughout the study.

The calves were randomly allocated to one of three experimental treatments using random number generator function in Microsoft Excel (Version 2019, Microsoft Corp., Redmond, WA, USA). The experimental treatments were 1) milk without any fortification or additives (CON), 2) milk fortified with 1 g/d of an advanced chelated trace minerals supplement ([BON] Bonzaplex7; Sodour Ahrar Shargh Co., Tehran, Iran), and 3) milk fortified with trace minerals from inorganic sources at levels equivalent to the BON treatment (IOR). The BON treatment followed the manufacturer’s recommendations, providing each calf daily with 51 mg Zn, 28 mg Mn, 18 mg Cu, 8 mg Fe, 1.7 mg Co, 0.5 mg Cr, and 0.3 mg Se. This mineral supplement is synthesized using patented chelate compound technology [21], which employs specific organic acids that undergo controlled polymerization to form a crystalline structure. The resulting Metal Organic Framework selectively incorporates mineral elements within its pores based on ionic radii specificity. The framework is constructed using a precise ratio of four aliphatic organic acids, including formic, propionic, citric, and tartaric acids, to create a unique porous crystalline matrix with defined binding properties. For IOR treatment, the inorganic sources used to fortify the milk were 159 mg zinc sulfate (32% elemental Zn), 82 mg manganese sulfate (34% elemental Mn), 90 mg copper sulfate (20% elemental Cu), 42 mg iron sulfate (19% elemental Fe), 9 mg cobalt Sulfate (19.5% elemental Co), 2.5 mg chromium chloride (19.5% elemental Cr), and 1 mg sodium selenite (42% elemental Se). The quantities of each inorganic compound were calculated to provide the same levels of zinc, manganese, copper, cobalt, selenium, iron, and chromium as the 1 g/d dosage of the advanced chelated trace minerals supplement in the BON treatment. The experimental trace minerals supplements were mixed daily with morning milk meal of each calf.

### Measurements

Calves were weighed at birth and every two weeks before morning milk feeding. Average daily gain (ADG) was estimated as the slop of a linier regression by plotting body weight against time, as described by Lancaster et al [28]. Starter intake was measured as a difference between the amount offered and refusals, and average daily starter intake (ADSI) was calculated for the periods from d 0 to 28, d 28 to 42, d 42 to 56, d 56 to 70, as well as throughout the pre-weaning period. Body measurements including hip height, wither height, body length, heart girth, and hip width were recorded on d 3 and 70 of age. Rectal temperature was taken daily from d 2 to 21 of age at 07:00 AM. Medical interventions for the calves were administered according to veterinary recommendations. During the first 21 d of life, all instances of intravenous fluid therapy, antibiotic injections, or both were recorded and analyzed as medical treatments. Fecal scores were monitored daily by trained technicians, using a scale from 1 (normal) to 4 (watery). The fecal consistency index (FCI) was calculated using the following formula [29].


$$\text{FCI}=\frac{\left(\text{DS}1\times 1)+(\text{DS}2\times 2)+(\text{DS}3\times 3)+(\text{DS}4\times 4\right)}{\text{TD}\times 4}$$

Where DS1, DS2, DS3, DS4 = number of days with fecal scores 1, 2, 3, 4, respectively, and TD = total number of days evaluated (21 d). This index provides an integrated assessment of fecal fluidity over the scoring period, with higher values indicating greater fecal looseness, potentially reflecting diarrhea in calves and serving as a key indicator of gastrointestinal health.

### Sample collection and analysis

Blood samples were collected from the calves on d 3, 15, and 70 of age before their morning milk feeding. The blood was centrifuged at 3,000×g for 10 minutes to separate the serum, which was then stored at −20°C until analysis. Serum analyses were conducted using commercial kits (Pars Azmoon, Tehran, Iran) according to the manufacturer’s instructions to determine the concentrations of glucose (Glu), blood urea nitrogen (BUN), triglycerides (TG), cholesterol (Chol), total protein (TP), albumin (Alb), aspartate aminotransferase (AST), and alanine aminotransferase (ALT). The concentration of globulins (Glob) was calculated by subtracting the Alb concentration from the TP concentration, while the Alb-to-globulin ratio (A/G) was calculated as the ratio of Alb to Glob concentrations.

To evaluate nutrient digestibility, fecal samples were collected from all calves via rectal palpation at four different time points over a four-days period: d 66 (8:00 AM and 12:00 PM), d 67 (9:00 AM and 1:00 PM), d 68 (10:00 AM and 2:00 PM), and d 69 (11:00 AM and 3:00 PM) of age. During these same four days, samples of the starter feed as well as the refusals were also collected. The fecal, starter feed, and refusal samples were dried at 70°C for 48 h to determine dry matter (DM) content. Subsequently, the dried samples were ground to pass through a 2 mm screen, and subsamples were subjected to chemical analyses conducted in triplicate.

Organic matter (OM) content was determined by ashing the samples at 600°C for 6 h. Neutral detergent fiber (NDF) and acid detergent fiber (ADF) were analysed following the method described by Van Soest et al [30]. NDF was measured without the use of α-amylase and sodium sulfite, and with excluding residual ash. Acid-insoluble ash was used as an internal marker to calculate nutrient digestibility coefficients, following the procedure described by Block et al [31].

### Statistical analysis

All data were screened for normality using the UNIVARIATE procedure of SAS (SAS 9.4, SAS Institute Inc., Cary, NC, USA). Data were analyzed using the MIXED procedure of SAS (SAS 9.4, SAS Institute Inc.) with a completely randomized design. For body weight (BW), ADSI, and blood metabolites, time was included as a repeated measure. The statistical model included the fixed effects of treatment, time, and their interaction, with the individual calf as the experimental unit. Body measurements, rectal temperature, FCI, and apparent total tract digestibility (ATTD) data were analyzed using the same model but without the effects of time and interaction. Birth BW (for BW and ADG), mid-test metabolic BW (for ADSI), and ADSI during the fecal sampling period (for ATTD) were included in the model as covariates. For body measurements and blood metabolites, baseline values measured on d 3 of age were also used as covariates. Two variance-covariance structures (autoregressive type 1 and compound symmetry) were evaluated to account for repeated measurements. The final structure was selected based on Schwarz’s Bayesian information criterion, which provided the best model fit [32]. This approach effectively handled both unbalanced data and individual variations over time. Data on medical treatments data were analyzed with a mixed-effects logistic regression model using PROC GLIMMIX procedure of SAS, where experimental treatment was included as a fixed effect and calf as a random effect. Least squares means were separated using the PDIFF statement p≤0.05. Statistical significance was declared at p<0.05, whereas results with 0.05<p≤0.1 were considered to indicate a trend towards significance.

## RESULTS

As a result of milk fortification with trace minerals, calves in the BON and IOR groups consumed, on average, more trace minerals than CON calves (Table 2). Two calves (1 male and 1 female) in the CON group succumbed to severe diarrhea and were euthanized, leading to their exclusion from subsequent data analysis. While this reduced our sample size, including their pre-mortality data could have introduced outliers from severely compromised physiological states, potentially distorting our understanding of typical treatment responses. Our reported results therefore reflect outcomes in calves that maintained sufficient health to complete the trial. This analytical approach, though conservative, provides more reliable insights into the typical physiological responses to mineral supplementation under normal conditions.

### Growth performance, starter intake, and skeletal development

**Figure 1:**
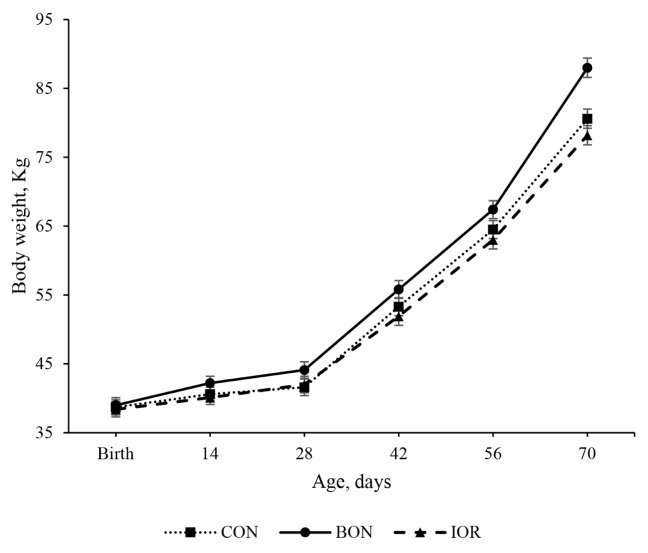
Body weight of calves receiving different experimental treatments throughout the study period. CON, milk without any fortification; BON, milk fortified with 1 g/d of an advanced chelated trace minerals supplement; IOR, milk fortified with trace minerals from inorganic sources at levels equivalent to the BON.

**Figure 2:**
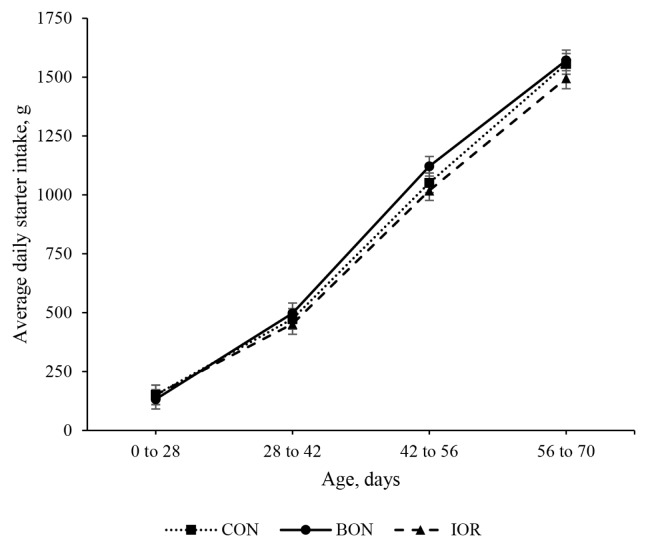
Average daily starter intake of calves receiving different experimental treatments throughout the study period. CON, milk without any fortification; BON, milk fortified with 1 g/d of an advanced chelated trace minerals supplement; IOR, milk fortified with trace minerals from inorganic sources at levels equivalent to the BON.

Significant differences in BW were observed among the experimental treatments, with the BON group exhibiting higher values (p = 0.027) compared to both the CON (88.0 vs. 80.6 kg; 95% CI: 4.7 to 10.1) and IOR (88.0 vs. 78.2 kg; 95% CI: 7.1 to 12.5) groups (Table 3). As shown in Figure 1, the effect of the advanced chelated trace mineral supplement on BW was time-dependent, with significant differences emerging from d 42 of age. Furthermore, ADG (p = 0.102) and feed conversion efficiency (FCE; p = 0.060) tended to be greater in the BON group compared to the other two groups. The statistical analysis did not show significant differences among the experimental groups for ADSI. However, ADSI in the BON calves was numerically higher than the CON and IOR groups from week 7 until weaning (Figure 2).

**Figure 3:**
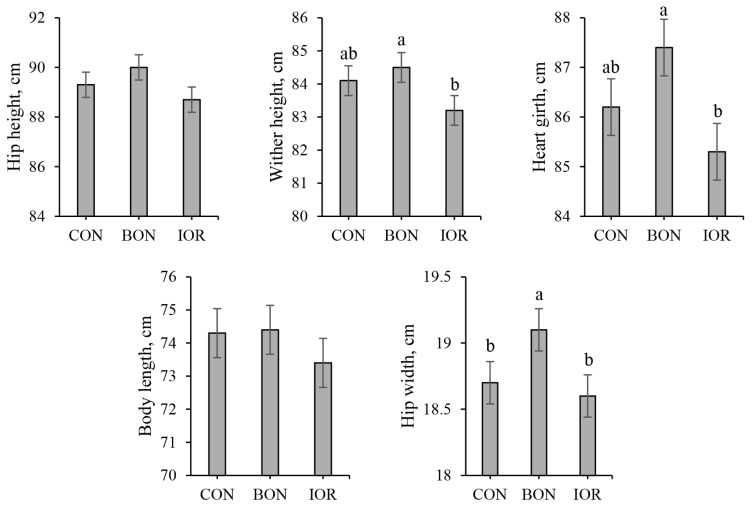
Body measurements of calves receiving different experimental treatments. CON, milk without any fortification; BON, milk fortified with 1 g/d of an advanced chelated trace minerals supplement; IOR, milk fortified with trace minerals from inorganic sources at levels equivalent to the BON. ^a,b^ Different letters indicate significant differences between mean values (p<0.05).

Figure 3 presents the body measurements of calves. No significant differences were observed among the treatments for hip height and body length. However, both wither height and heart girth were significantly higher in BON group compared to IOR (p<0.05). Additionally, a significant difference was observed among the groups for hip width, with the BON calves exhibiting significantly higher values than the CON and IOR calves. Although the calves in the IOR group showed lower body measurement values, the differences between the CON and IOR groups were not statistically significant (p> 0.05).

### Rectal temperature, medical treatment, and fecal consistency index

**Figure 4:**
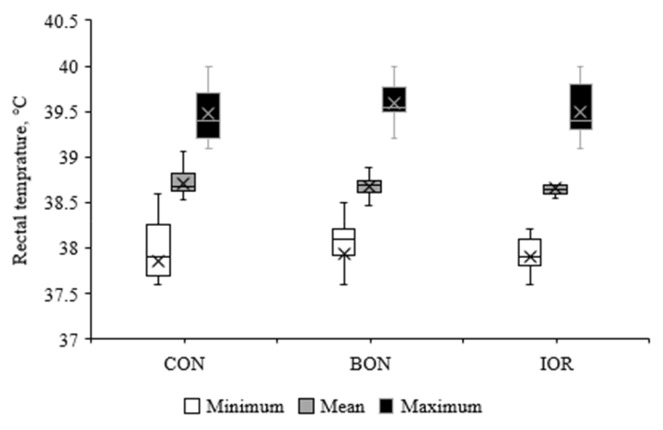
Rectal temperatures of calves measured during the first 21 days of life under different experimental treatments. CON, milk without any fortification; BON, milk fortified with 1 g/d of an advanced chelated trace minerals supplement; IOR, milk fortified with trace minerals from inorganic sources at levels equivalent to the BON.

**Figure 5:**
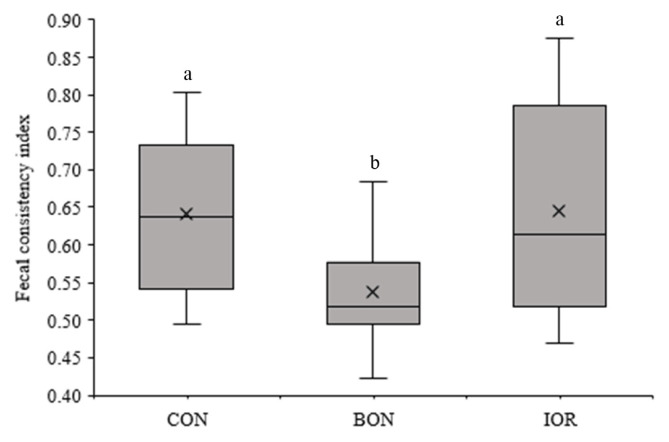
Fecal consistency index of calves measured during the first 21 days of life under different experimental treatments. CON, milk without any fortification; BON, milk fortified with 1 g/d of an advanced chelated trace minerals supplement; IOR, milk fortified with trace minerals from inorganic sources at levels equivalent to the BON. ^a,b^ Different letters indicate significant differences between mean values (p<0.05).

**Figure 6:**
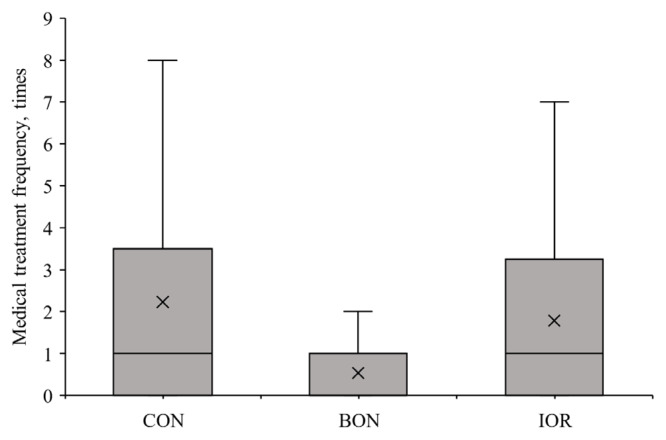
Frequency of medical treatment applied to calves during the first 21 days of life under different experimental treatments. CON, milk without any fortification; BON, milk fortified with 1 g/d of an advanced chelated trace minerals supplement; IOR, milk fortified with trace minerals from inorganic sources at levels equivalent to the BON.

Figure 4 illustrates the mean, minimum, and maximum rectal temperatures of calves. No significant differences were detected among the groups for rectal temperature (p>0.05). However, the calves in the BON group exhibited a lower FCI compared to the CON (0.55 vs. 0.62; 95% CI: −0.05 to −0.09) and IOR (0.55 vs. 0.65; 95% CI: −0.08 to −0.12) groups (p = 0.021; Figure 5). Moreover, the frequency of medical treatments, was also decreased in the BON calves compared to the other two groups (p = 0.103; Figure 6).

### Blood metabolites and nutrient digestibility

The results of blood metabolites are shown in Table 4. Treatment significantly affected serum AST concentration (p = 0.015) and showed tendencies to influence BUN (p = 0.101), Alb (p = 0.082), A/G ratio (p = 0.083), and ALT (p = 0.078) concentrations. Specifically, CON calves exhibited higher BUN and ALT levels, whereas BON calves demonstrated the highest Alb concentrations and A/G ratios, accompanied by the lowest Glob values. The main effects of time on Glu, A/G ratio, TG, Chol, and AST were significant with higher values observed on d 70 compared to d 15 (p<0.05). Moreover, the serum concentrations of BUN, TP, and Glob were lower on d 70 than on d 15 (p<0.05).

Significant interactions were detected between treatment and time for several blood metabolites. The serum concentration of BUN at 15 d of age was significantly higher in the IOR group compared to the CON and BON groups (p<0.05), while no significant differences were observed on d 70. A similar pattern was also observed for Glob concentration and the A/G ratio, with significant differences (p<0.05) among groups only present on d 15 but not on d 70. Additionally, Alb concentration on d 15 was higher for the BON group compared to the other two groups (p<0.05). However, on d 70, the difference between the BON and IOR groups for Alb concentration was not significant, and both groups had lower Alb levels than the CON group (p<0.05). Differences among the groups for the A/G ratio and ALT were also significant on d 15 but not at 70 d of age.

The results of nutrient digestibility are presented in Table 5. No significant differences were observed among the experimental group for ATTD of DM, OM, NDF, and ADF.

## DISCUSSION

Trace mineral requirements of calves are typically met through the dam’s milk and solid feed intake in natural settings. However, in intensive dairy production systems where calves are separated from their dams and fed milk or milk replacer, trace minerals are commonly supplemented in starter feed or as a component of milk replacer [33]. Due to the tight regulation of trace mineral transfer from maternal diet to milk, the milk itself may be inadequate in some trace mineral content [34]. Additionally, dairy calves in intensive production systems often face numerous environmental stressors that may increase their trace mineral requirements [35,36].

Although trace mineral requirements and dietary supplementation recommendations for dairy cows are well established [37], optimal trace mineral requirements for neonatal calves remain inadequately defined. Previous studies have reported that milk supplementation with specific trace minerals, such as zinc and selenium, may reduce the incidence of diarrhea in calves and have positive impacts on growth and health [16,18,38–40]. However, research investigating comprehensive multiple trace minerals supplementation in milk is limited. The present study offers novel insights by examining a specific chelation technology that may enhance mineral bioavailability through its distinctive molecular architecture. Our selection of seven minerals was guided by their documented synergistic roles in critical physiological processes; copper and zinc for immune function, selenium and vitamin E interactions for antioxidant defense, manganese and chromium for growth and metabolism, and iron and cobalt for hematopoiesis [37,41]. This mineral profile aligns with standard supplementation practices in commercial dairy operations while addressing key physiological requirements.

The organic form of trace minerals in the diet of growing livestock has been shown to have a positive effect on growth performance due to superior bioavailability compared to inorganic forms [42]. Chelated or complexed minerals form stable soluble molecules that are better absorbed because their metal ions are bound to organic substances (amino acids, peptides, or polysaccharides) and are absorbed through ion-bound organic ligand pathways, minimizing interference from other molecules. However, bioavailability among organic trace minerals varies depending on ligand type, solubility, and production conditions [43,44]. While the precise mechanisms of the chelated compound technology used in our supplements remain to be fully elucidated, previous studies have demonstrated its superior effectiveness compared to inorganic forms for specific physiological responses. For instance, Mousavi-Haghshenas et al [26] recently reported that dietary supplementation of starter feed with an advanced chelated trace minerals supplement (similar to that used in our experiment) increased growth rates in low birth weight calves. Our findings corroborate these results, demonstrating positive effects on BW and body measurements when chelated trace minerals were added to milk. The impact of milk fortification with trace minerals on newborn ruminant growth shows variation across studies. While some research has documented improved growth rates with organic trace mineral supplementation [16,40], others have found no significant effects [45]. Evidence suggests that growth responses to milk fortification with trace minerals may be influenced by multiple factors, including animal sex [38,46] and plan of nutrition [10]. The trend toward improved ADG and FCE observed in our study may be partially attributed to the mixed-gender composition of the experimental groups. Furthermore, optimizing supplement concentrations in milk or adjusting trace mineral ratios within the supplement could potentially yield more pronounced effects on ADG and FCE in future studies.

Interestingly, supplementation with equivalent amount of trace minerals from inorganic sources not only failed to improve growth performance but also caused decreases in growth rate compared to calves receiving un-supplemented milk (i.e. CON calves). While both BON and IOR groups received trace minerals above the recommended levels (Table 2), the responses differed markedly. Although trace minerals are essential for calves, excessive dietary intake can negatively impacts health and productivity, primarily due to interactions and competition among minerals [37,47]. Organic trace minerals typically demonstrate reduced susceptibility to these antagonistic interactions, potentially enhancing their bioavailability and effectiveness [42]. The negative effects observed in the IOR treatment may also be partially attributed to elevated sulfur levels, as the inorganic minerals were predominantly supplied as sulphate salts. Sulfur is well documented as a major antagonist of trace mineral absorption and utilization in ruminants [48]. Our findings, however, indicated that the response of calves to high levels of trace minerals depends on the mineral source. The superior performance of calves receiving advanced chelated trace minerals, even at levels exceeding recommendations, indicates enhanced bioavailability and utilization compared to inorganic sources. Future research is needed to elucidate the specific mechanisms underlying the improved efficiency of chelated trace minerals compared to their inorganic counterpart.

Increased solid feed intake during the weaning transition is crucial for rumen development, as it provides physical stimulation and promotes volatile fatty acid production, ultimately leading to enhanced starter intake and improved post-weaning weight gain [49]. Additionally, improved nutrient digestibility often indicates a more developed and functional rumen [50]. However, in the present study, we observed no significant differences among experimental groups in either solid feed intake or ATTD of nutrients. The minimal effect of milk fortification with trace minerals on starter intake aligns with findings from previous studies [10,16,40]. Given the critical importance of post-weaning starter intake for dairy cattle development, future research should focus on examining the long-term effects of milk fortification with the chelated trace minerals on post-weaning feed intake and digestibility patterns.

The results of the present study demonstrate that supplementation with advanced chelated trace minerals significantly reduced both the incidence of diarrhea symptoms and the need for medical treatment in calves during their first 21 d of life. However, rectal temperature, a crucial indicator of inflammatory responses and potential infections [51], did not differ among the experimental groups. These parameters are widely recognized indicators of calf health status. Specifically, fecal consistency scores help assess digestive health and identify early signs of enteritis [29]. while the frequency of medical treatments reflects overall disease resistance [9]. Trace minerals, particularly copper, zinc, and selenium, play crucial roles in various physiological processes, including immune function, antioxidant defense, and gastrointestinal health [10,40,52]. These elements are essential for reducing free radicals and bolstering the animal’s immune system [41]. Additionally, dietary trace mineral supplementation can significantly influence gut microbiota development, which begins at birth and plays a crucial role in establishing host defense mechanisms [53–55]. The early-life gut microbial community has lasting effects on animal health through multiple mechanisms. A well-established gut microbiota forms a protective barrier on intestinal epithelial cells and helps prevent pathogen colonization through competitive exclusion and nutrient competition [55,56]. This interaction between trace minerals and gut microbiota development is particularly important during the pre-weaning period when the digestive system is rapidly developing [55]. Our observations of reduced diarrhea incidence in calves supplemented with advanced chelated trace minerals align with prior studies, which indicate that trace mineral supplementation can positively influence intestinal health and reduce enteric diseases in young animals [13,31,39]. For instance, the efficacy of zinc supplementation in reducing diarrhea symptoms has been well-documented. Previous studies reported that feeding calves in the first 14 d of life with milk supplemented with 80 mg/d of zinc, either from inorganic sources (zinc sulfate or zinc oxide) or zinc-methionine, reduced diarrhea symptoms [16,38–40]. In our study, the amount of zinc added to the milk in both the BON and IOR groups was 51 mg/d. Interestingly, despite the lower zinc content, the advanced chelated trace minerals supplement demonstrated superior effectiveness in reducing diarrhea incidence compared to the inorganic form. This effect may potentially be attributed to the enhanced absorption and utilization of trace minerals from the advanced chelated source. Moreover, the positive effect observed in the BON treatment may be partially attributed to the organic acids used in the chelated trace minerals supplement. Organic acids have been shown to have beneficial effects on gut health, including the modulation of gut microbiota and enhancement of nutrient digestibility [57]. The synergistic effect of chelated trace minerals and organic acids may have contributed to the improved intestinal health and reduced diarrhea incidence observed in our study.

Higher serum TP levels observed in the CON and IOR groups on d 15 of age were associated with lower Alb concentrations, higher Glob concentrations, and consequently, a lower A/G ratio compared to the BON group. Consistent with our findings, previous research has demonstrated that increased plasma TP correlates with elevated globulin levels and can be modulated by dietary mineral supplementation. For instance, milk enrichment with Se and vitamin E increased plasma TP and Glob levels in newborn goat kids [46]. Similarly, Mudgal et al [58] reported elevated Glob levels accompanied by reduced Alb concentrations in the plasma of 8 to 9 month old buffalo calves supplemented with inorganic Se and Cu, though this effect was not observed in 15-month-old animals [59]. The lower A/G ratio observed in our study likely reflects reduced infection rates in BON calves compared to the CON group, as evidenced by lower FCI and fewer required medical treatments. During infectious conditions, animals typically experience increased Glob production accompanied by a concurrent decrease in blood Alb concentrations. Previous research has demonstrated a significant inverse correlation between A/G ratio and elevated somatic cell counts in lactating cows [60]. In our study, we observed negative correlations between the A/G ratio and both FCI and medical treatment frequency. These findings suggest that milk fortification with advanced chelated minerals may help prevent infectious diseases that trigger increased Glob production in pre-weaned calves, an effect not observed with inorganic trace mineral sources. Moreover, the activity of AST was higher in the IOR compared to the CON and BON groups. On d 15 of age, the IOR group also showed higher levels of ALT activity than the other two groups. Both AST and ALT enzymes are well established biomarkers of liver function, with elevated levels typically indicating hepatic tissue damage [61]. Extensive research has demonstrated that dietary trace mineral supplementation can significantly influence the activity of hepatic enzymes [62]. Research evidence suggests that disruptions in metal homeostasis, whether through impaired transport mechanisms or compromised hepatic detoxification pathways, can lead to metal accumulation and subsequent hepatotoxic effects through multiple cellular mechanisms [63]. However, the literature shows heterogeneous responses to different mineral sources. Several studies have reported significant variations in ALT and/or AST activities between chelated and inorganic mineral source [64–66], while others have found no differences in enzyme activities between sources [58,67,68]. These inconsistent findings might be attributed to variations in experimental conditions, animal species, mineral concentrations, specific chelation technologies used, or interactions with other dietary components. Further research using more sensitive biomarkers of liver function, along with histological examination, would help clarify the specific effects of different mineral sources on hepatic health.

## CONCLUSION

In conclusion, our study demonstrates promising effects of advanced chelated trace minerals on dairy calf development when administered through milk, with notable improvements in specific growth parameters and health indicators. Specifically, we observed an 11% reduction in diarrhea incidence and 9% improvement in BW compared to control calves. The findings also indicated that mineral form, not merely supplementation level, plays a crucial role in early calf development. For practical implementation, our findings suggest that dairy farmers could consider incorporating chelated minerals into their existing feeding protocols, particularly during the critical pre-weaning period. While the initial cost of chelated minerals may be higher than conventional supplements, the observed reductions in medical treatments and improved growth rate suggest potential economic benefits that merit further investigation. Future research should also address several key areas: First, multi-breed studies across diverse management systems would help validate these findings’ broader applicability. Second, long-term follow-up studies are needed to assess impacts on lifetime productivity, including postweaning growth performance, first lactation performance, and reproductive efficiency. Third, investigation of optimal supplementation levels for different age groups and production stages would provide more precise feeding recommendations. Additionally, mechanistic studies examining gut microbiome composition, immune function markers, and mineral absorption pathways would help explain the observed variations in response to chelated minerals and potentially identify early biomarkers for supplementation effectiveness. From a broader industry perspective, these findings contribute to the ongoing evolution of precision nutrition in dairy farming. However, the practical implementation of chelated mineral supplementation programs requires careful consideration of farm-specific factors, including existing mineral status of feeds, water quality, and economic constraints. By addressing these aspects in future research while building upon our current findings, we can develop more refined, evidence-based nutritional strategies that optimize both animal health and farm profitability.

## Figures and Tables

**Table 1:** Ingredients and chemical composition of the starter diet

Ingredients (g/kg DM)	Contents
Alfalfa hay	70
Wheat straw	30
Barley grain	135
Corn grain	425
Soybean meal	238
Wheat milling	42.5
Salt	6.8
Sodium bicarbonate	12.75
Calcium carbonate	4.25
Magnesium oxide	2.55
Dicalcium phosphate	4.25
Mineral premix^1)^	12.75
Vitamin premix^2)^	14.45
Bentonite	1.7
Analyzed chemical composition (%)	
DM	88.4
CP	19.4
NDF	24.1
ADF	18.4
Calculated supplemental trace minerals content, ppm	
Zn	143.4
Mn	108.4
Cu	40.8
Co	1.3
Se	1.0

1) Mineral premix contained (per kg): Ca 290 g, Mg 15.3 g, Zn 11,250 mg, Mn 8,500 mg, Cu 3,200 mg, I 190 mg, Co 105 mg, and Se 80 mg.

2) Vitamin premix contained (per kg): Vit A 1,200,000 IU, Vit D3 250,000 IU, Vit E 10,000 IU, Monensin 3 g, Biotin 200 mg.

DM, dry matter; CP, crude protein; NDF, neutral detergent fiber; ADF, acid detergent fiber.

**Table 2:** Average daily trace mineral intake of calves from supplemental sources

Trace mineral (mg/d)	Treatments^1)^		

	CON	BON	IOR
Zn	106.0	155.9	153.1
Mn	80.1	109.5	105.2
Cu	30.2	48.7	47.0
Fe	NS	8.0	8.0
Co	0.98	2.71	2.65
Cr	NS	0.50	0.50
Se	0.75	1.07	1.03

1) CON, milk without any fortification; BON, milk fortified with 1 g/d of an advanced chelated trace minerals supplement; IOR, milk fortified with trace minerals from inorganic sources at levels equivalent to the BON.

NS, non-supplemented.

**Table 3:** Effect of trace minerals fortification in milk on growth performance of pre-weaned dairy calves

Items	Treatments^1)^			SEM	p-value		
	
	CON	BON	IOR		Treatment	Time	Treatment×Time
IBW (kg)	38.7	39.0	38.4	1.1	0.709	-	-
BW (kg)	80.6^b^	88.0^a^	78.2^b^	1.4	0.027	< 0.01	0.041
ADG (kg)	0.57	0.64	0.55	0.03	0.102	-	-
ADSI (kg)	0.81	0.83	0.78	0.41	0.163	< 0.01	0.774
FCE (kg/kg)	0.72	0.80	0.73	0.104	0.060	-	-

1) CON, milk without any fortification; BON, milk fortified with 1 g/day of an advanced chelated trace minerals supplement; IOR, milk fortified with trace minerals from inorganic sources at levels equivalent to the BON.

a,b Values with different superscripts within a row are significantly different (p≤0.05).

SEM, standard error of the mean; IBW, initial (birth) body weight; BW, body weight; ADG, average daily gain; ADSI, average daily starter intake; FCE, feed conversion efficiency (kg ADG / kg ADSI).

**Table 4:** Effect of trace minerals fortification in milk on the concentrations of blood metabolites in pre-weaned dairy calves

Treatments^1)^	Items									

	Glu (mg/dL)	BUN (g/dL)	TP (g/dL)	Alb (g/dL)	Glob (g/dL)	A/G	TG (mg/dL)	Chol (mg/dL)	AST (U/L)	ALT (U/L)
Day 15 of age										
CON	73.0	11.2^b^	5.5	2.9^b^	2.6^a^	1.2^b^	12.8	51.2	28.3	5.5^c^
BON	78.7	11.9^b^	4.9	3.2^a^	1.9^b^	1.7^a^	11.5	50.2	23.5	6.7^bc^
IOR	74.8	16.7^a^	5.6	3.0^b^	2.6^a^	1.2^b^	10.2	48.7	49.3	15.7^a^
SEM	4.81	0.69	0.15	0.05	0.12	0.06	1.78	4.10	2.57	1.18
Day 70 of age										
CON	94.7	10.0^bc^	5.2	3.3^a^	1.9^b^	1.7^a^	30.0	86.8	46.2	10.3^b^
BON	90.5	9.1^c^	4.7	2.9^b^	1.7^b^	1.7^a^	32.2	82.7	40.5	10.0^b^
IOR	95.0	9.5^c^	4.7	2.9^b^	1.8^b^	1.7^a^	28.7	107.8	49.3	9.5^b^
SEM	6.01	0.42	0.21	0.05	0.12	0.07	2.29	7.12	3.18	1.82
p-value										
Treatment	0.989	0.101	0.118	0.082	0.139	0.083	0.751	0.303	0.015	0.078
Time	0.029	<0.01	0.040	0.717	<0.01	<0.01	<0.01	<0.01	<0.01	0.703
Treatment × Time	0.838	0.046	0.304	0.026	0.022	0.042	0.779	0.124	0.669	0.041

1) CON, milk without any fortification; BON, milk fortified with 1 g/d of an advanced chelated trace minerals supplement; IOR, milk fortified with trace minerals from inorganic sources at levels equivalent to the BON.

a–c Values with different superscripts within a column are significantly different (p≤0.05).

Glu, glucose; BUN, blood urea nitrogen; TP, total protein; Alb, albumin; Glob, globulin; A/G, albumin to globulin ratio; TG, triglycerides; Chol, cholesterol; AST, aspartate aminotransferase; ALT, alanine aminotransferase; SEM, standard error of the mean.

**Table 5:** Effect of trace minerals fortification in milk on apparent total tract digestibility of nutrient in pre-weaned dairy calves

Items	Treatments^1)^			SEM	p-value

	CON	BON	IOR		
DM	75.6	75.3	74.9	1.49	0.938
OM	77.2	76.9	76.5	1.60	0.949
NDF	38.4	38.5	37.0	1.19	0.617
ADF	33.9	37.7	34.7	2.71	0.573

1) CON, milk without any fortification; BON, milk fortified with 1 g/day of an advanced chelated trace minerals supplement; IOR, milk fortified with trace minerals from inorganic sources at levels equivalent to the BON.

SEM, standard error of the mean; DM, dry matter; OM, organic matter; NDF, neutral detergent fiber; ADF, acid detergent fiber.
